# The impact of pericardial approach and myocardial protection onto postoperative right ventricle function reduction

**DOI:** 10.1186/s13019-018-0726-5

**Published:** 2018-06-05

**Authors:** Marco Zanobini, Claudia Loardi, Paolo Poggio, Gloria Tamborini, Fabrizio Veglia, Alessandro Di Minno, Veronika Myasoedova, Liborio Francesco Mammana, Raoul Biondi, Mauro Pepi, Francesco Alamanni, Matteo Saccocci

**Affiliations:** 1 Department of Cardiac Surgery, Centro Cardiologico Monzino IRCCS, University of Milan, Via Parea, 4, 20138 Milan, Italy; 2 Heart Center, University Hospital of Zürich, University of Zürich, Zürich, CH Switzerland

**Keywords:** Mitral valve, Valve repair, Minimally invasive surgery, Right ventricle, Cardioplegia, Echocardiography

## Abstract

**Background:**

The reduction of RV function after cardiac surgery is a well-known phenomenon. It could persist up-to one year after the operation and often leads to an incomplete recovery at follow-up echocardiographic control. The aim of the present study is to analyze the impact of different modalities of pericardial incision (lateral versus anterior) and of myocardial protection protocols (Buckberg versus Custodiol) onto postoperative RV dynamic by relating two- and three-dimensional echocardiographic parameters in patients undergoing mitral valve repair through minimally invasive or traditional surgery approach.

**Methods:**

We have analyzed 44 consecutive patients with severe degenerative mitral regurgitation who underwent mitral reparation with different surgical approach and cardioplegia type: Group 1 (17 pts): sternotomy with Buckberg cardioplegia protocol; Group 2 (10 pts): sternotomy with Custodiol cardioplegia; Group 3 (17 pts): mini-invasive surgery with Custodiol cardioplegia. Two-dimensional transthoracic echocardiography was performed pre- and 6 months post-surgery to evaluate RV function by tricuspid annular plane systolic excursion (TAPSE).

**Results:**

All patients underwent successful and uneventful. A postoperative TAPSE reduction was found in all groups. However, mini-invasive patients experienced a significant reduced variation versus traditional surgery.

**Conclusions:**

Mini-invasive mitral repair, with lateral incision of pericardium, reduces postoperative TAPSE fall, while cardioplegia protocol fails to have an impact onto longitudinal RV function. In our study, the RV seems to experience a clinically irrelevant geometrical modification too, whose entity appears to be less evident in case of lateral pericardial approach. These results could strengthen the use of minimally invasive approach also to preserve RV function.

## Background

Right ventricular function is widely known as a determinant of exercise capacity and as significant prognostic value in the evaluation of surgical outcome [1, 2]. During and immediately after cardiac surgery, it is known that there is a decrease of two-dimensional indexes of right ventricle systolic performance [3–5]. Recovery to basal values is often incomplete and an echocardiographic dysfunction can persist even at one year after surgery [6].

Physiopathology of right ventricle behavior following cardiac surgery is a largely debated issue and several hypotheses have been suggested: 1) myocardial hypothermia [7]; 2) cardiopulmonary bypass use [8]; 3) pericardial adhesions [9]. Pericardial opening need [10] and modality of cardioplegia delivery, retrograde cardiac protection seems to be less effective in preserving right ventricle function, [11] appear to be mainly involved.

Two-dimensional echocardiography represents the reference method for cardiac surgery patients’ follow up; however, regarding the evaluation of right ventricle performance, it has important limitations due to its particular anatomy. The recent introduction of 3-dimensional echocardiographic images allows a more complete evaluation of right ventricle contraction, showing that two-dimensional indexes decrease failed to be accompanied by concomitant parallel right ventricle three-dimensional functional changes [3].

Unfortunately, 3D echocardiograms can be performed only by highly specialized experienced operators. Indeed, 2D measurements like tricuspid annular plane systolic excursion (TAPSE) is still largely used in right ventricular evaluation.

The aim of the present study is to evaluate the impact of the type of pericardial incision (lateral versus anterior) in combination of different myocardial protection protocols onto postoperative right ventricular systolic function in order to further investigate the superiority of minimally invasive approach in mitral valve surgery.

## Methods

### Population and study protocol

All patients were enrolled in our Center and operated by the same surgeon. Written informed consent to participate in this observational study, which was approved by Centro Cardiologico Monzino Institutional Review Board, was obtained from all patients. The study protocol conforms to the ethical guidelines of the Declaration of Helsinki as reflected in a priori approval by the institution’s human research committee.

To achieve the aim of this study, we compared 2- and 3-dimensional echocardiographic parameters in patients undergoing minimally invasive or traditional (full sternotomy) mitral valve repair (MVR) focusing onto the impact of pericardial incision and cardioplegia protocol.

**Fig. 1:**
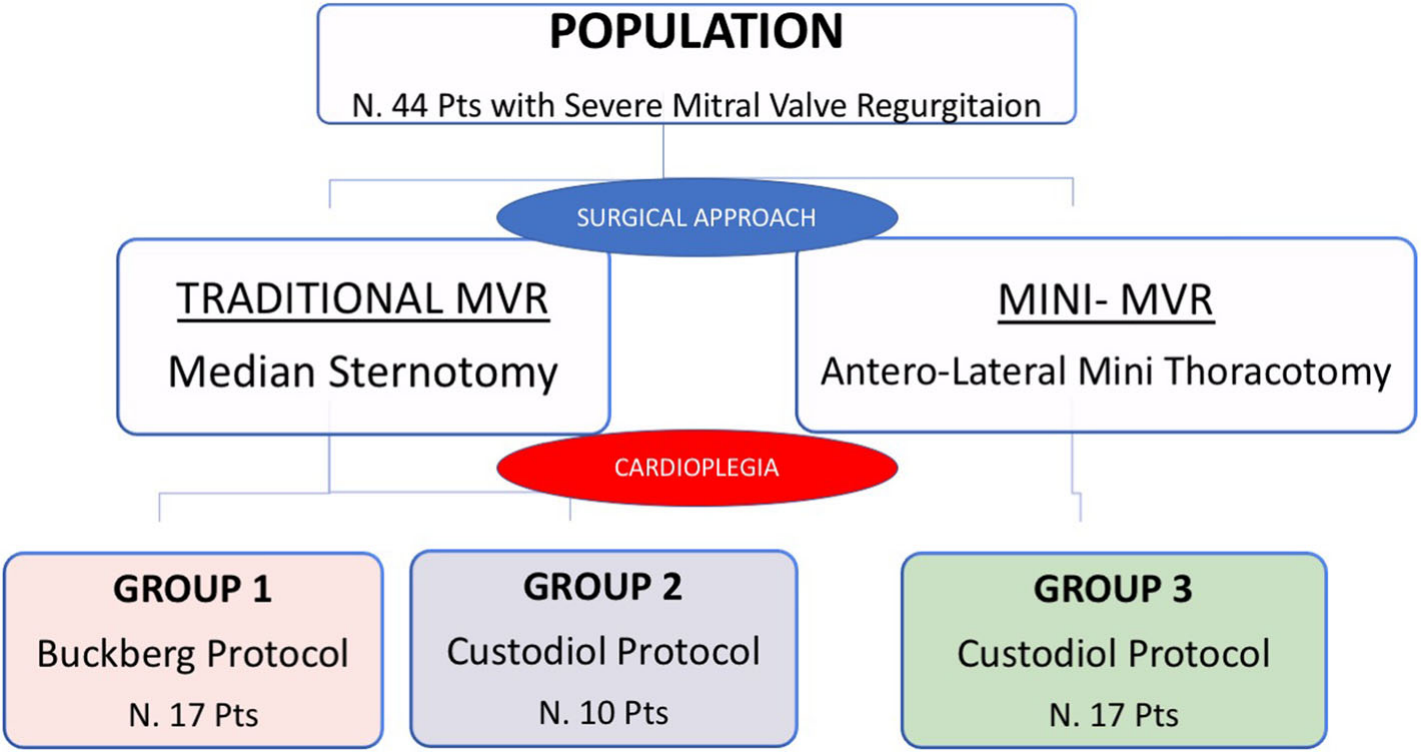
Design of the study

We retrospectively analyze data of 44 consecutive patients (mean age 54 ± 12 years; 34 males/10 females) with severe mitral regurgitation related to degenerative dysfunction due to mitral valve prolapse who underwent mitral valve reparation at our Center by the same Surgeon. We subdivided them in 3 groups according to surgical approach and cardioplegia type (Fig. 1):

1. Group 1: traditional sternotomy operation with Buckberg cardioplegia protocol (blood mixed antegrade-retrograde solution) – 17 patients
2. Group 2: traditional sternotomy operation with Custodiol cardioplegia (crystalloid antegrade administration) – 10 patients
3. Group 3: mini-invasive surgery (4 cm right antero-lateral thoracotomy) with Custodiol cardioplegia (crystalloid antegrade administration) – 17 patients

Mini-invasive mitral surgery tends to be performed with single shot cardioplegia protocol, like Custodiol, while Buckberg protocol seems to be more protective and versatile for traditional surgery. The design of the study permits to investigate independently surgical approach and cardioplegia making a comparison between groups with same pericardial access (group 1 and 2) but different myocardial protection and between groups with same cardioplegia protocol but different incision of the pericardium (group 2 and 3).

Clinical and echocardiographic baseline patients’ characteristics are shown in Table 1. Composition of cardioplegic solutions is reported in Table 2.

**Table 1:** Clinical and echocardiographic groups’ baseline characteristics

	Group 1 (*n* = 17)	Group 2 (*n* = 10)	Group 3 (*n* = 17)	*P* value
Age, y	54.8 ± 11.9	59.2 ± 10.2	50.9 ± 12.2	0.14
Male, n (%)	13 (76%)	7 (70%)	14 (82%)	0.18
BSA (m^2^)	1.9 ± 0.2	1.8 ± 0.2	1.8 ± 0.1	0.32
EuroSCORE 2^*^	0.98%	0.96%	0.95%	0.45
LVEF	60.4 ± 6.4	58.2 ± 4.1	64.5 ± 4.7	0.32
TAPSE (mm)	24.5 ± 4.8	27.4 ± 5.3	23.5 ± 4	0.17
SPAP (mmHg)	33.9 ± 4.8	32.3 ± 5.1	34.9 ± 2.7	0.83
RVEF (3D)	62.2 ± 8.9	60.5 ± 8.2	58.5 ± 6.1	0.56
RVSV (3D)	68.3 ± 23.9	54.1 ± 18.8	68 ± 13.1	0.21
RVESV (3D)	43.4 ± 22.5	34.7 ± 11.7	48.5 ± 11.6	0.34^Ɨ^
RVEDV (3D)	111.7 ± 42.8	88.8 ± 26.1	116.5 ± 19.8	0.18^φ^
MVP Type				
Posterior leaflet prolapse	17	9	17	
Anterior leaflet prolapse	2	1	1	

*EuroSCORE 2 denotes the European System for Cardiac Operative Risk Evaluation (2nd version)

^Ɨ^*p* = 0.04 Group 2 versus 3

^φ^*p* = 0.03 Group 2 versus 3

*BSA* Indicates body surface area, *LVEF* Left ventricular ejection fraction, *TAPSE* Tricuspid annular plane systolic excursion, *SPAP* Systolic pulmonary arterial pressure, *RVEF* Right ventricular ejection fraction, *RVSV* Right ventricular stroke volume, *RVESV* Right ventricular end-systolic volume, *RVEDV* Right ventricular end-diastolic volume, *MVP* Mitral valve prolapse

**Table 2:** Composition of cardioplegic solutions used in the study

Ingredient	Buckberg cold induction 4.1	Buckberg cold maintenance 4.1	Buckberg “hot shot”	Custodiol
Na^+^	140	140	140	15
K^+^	20	10	8	9
Mg^++^	13	9	6	4
Ca^++^	–	–	–	0.015
Hystidine	–	–	–	198
Tryptophan	–	–	–	2
Ketoglutarate	–	–	–	1
Mannitol	–	–	–	30
Glucose	6	6	8	–
pH	7.2	7.4	7.5	7.02–7.2

All ingredients are expressed in mmol/L

Exclusion criteria were persistent or paroxysmal atrial fibrillation, urgent intervention with hemodynamic instability, poor echocardiographic acoustic apical window, tricuspid regurgitation major than 1 degree (scale 1 to 4), concomitant surgery procedures or mitral valve replacement, major pulmonary diseases justifying a right ventricular dysfunction or pulmonary hypertension, previous cardiac surgery and preoperative reduced LV function (Ejection fraction < 40%). Two-dimensional (2D-) and three-dimensional (3D-) transthoracic echocardiography (TTE) was performed preoperative (24–48 h before surgery) and 6 months after surgical operation.

### Surgical procedures

Traditional MVR patients (group 1 and 2) underwent complete median sternotomy and anterior opening of the pericardium with a reversed T incision. Standard cardiopulmonary bypass (CBP) was implanted with ascending aortic cannulation and bicaval venous cannulation.

In Group 1 Buckberg cardioplegia was adopted, consisting in a three-phases myocardial protection protocol:

1. Cold induction: delivery of cold cardioplegic solution (8–12 °C) antegrade and retrograde for 2 min each until complete cardioplegic arrest was achieved (flow 200 ml/min, in hypertrophied hearts increase to 300 ml/min)
2. Reinfusions with cold blood cardioplegia: during aortic cross-clamping, multidose cold blood cardioplegia was applied at intervals of 20 min to maintain cardioplegic arrest and myocardial hypothermia. Infusions were delivered retrograde for 1 min (flow 200 ml/min)
3. Warm terminal reperfusion (“hot shot”): normothermic substrate-enriched blood cardioplegia was applied before aortic unclamping. The warm reperfusate was delivered via coronary sinus for 1 min and followed by a brief (20–30 s) retrograde administration of normothermic blood [12].

In Group 2 Custodiol protocol protection (single antegrade injection of crystalloid intracellular cold cardioplegia in 6–8 min) was employed.

Minimally invasive mitral valve repair (Group 3) was performed through a small (5 cm) right antero-lateral thoracotomy and with a lateral supra-phrenic pericardial incision. Peripheral CBP was implanted using femoral vessels for arterial and venous cannulation. Similarly to Group 2, a single antegrade dose of Custodiol cardioplegia was administered.

All interventions were performed in moderate hypothermia (32 °C) with a direct left atriotomy through Waterstone’s groove. Depending on patient’s mitral alteration we used different surgical repairing techniques always supported by annuloplasty with a prosthetic ring implantation (Table 3). In each group the pericardium was completely re-closed with a continuous suture.

**Table 3:** Intraoperative groups’ characteristics

	Group 1 (*n* = 17)	Group 2 (*n* = 10)	Group 3 (*n* = 17)	*P* value
CPB time (min)	113 ± 17	105 ± 18	131 ± 23	0.21
Cross-clamp time (min)	95 ± 13	93 ± 12	112 ± 12	0.24
Complete prosthetic semi-rigid ring	3	2	2	0.32
Incomplete band	14	8	15	0.25
Annular plication	1	0	0	0.43
Quadrangular resection	6	4	7	0.44
Triangular resection	9	5	9	0.28
Sliding-plasty	5	4	6	0.12
Artificial chordae positioning	2	1	1	0.23
Papillary muscle plication	1	0	0	0.34

*CPB* Indicates cardio-pulmonary bypass

### Echocardiographic measurements

We performed a complete standard M-mode and two-dimensional echocardiographic examinations using a Philips ultrasound system (iE33, Andover, MA, USA) and an S5–1 sector array probe. LV end-diastolic and end-systolic volumes, as well as biplane ejection fraction were measured in apical four- and two chamber views with the area–length method. Systolic pulmonary arterial pressure was non-invasively acquired using Doppler echo method from the systolic right ventricle–right atrial gradient, calculated from the systolic trans-tricuspid regurgitant flow peak velocity by the modified Bernoulli equation. Right atrial (RA) pressure was derived by means of the inferior vena cava (IVC) collapsibility index measured from the subcostal view [13]. To estimate tricuspid annular plane systolic excursion (TAPSE), defined as the difference in the displacement of the right ventricle base from end-diastole to end-systole, from the apical four-chamber view, the M-mode cursor was positioned at the junction of the tricuspid valvular plane with the right ventricle free [14, 15].

3D- Real-time TTE was performed during same echocardiographic session utilizing an X3–1 matrix array probe. The 3D-measurement were acquired using ‘full volume’ mode from the apical view, adapted to improve the visualization of the right ventricular chamber. We registered Two datasets for each patient. To perform offline post-processing and three-dimensional reconstruction we used a commercially available dedicated system (Echo View, Tom Tec Imaging Inc., Munich, Germany) equipped with a four-dimensional right ventricle analysis software [16].

### Statistical analysis

Data were managed in Microsoft Excel 2016 and analyzed with SPSS 22.0 software (SPSS, Inc., Chicago, IL) and SAS system (SAS Institute Inc., Cary, NC). Each echocardiographic parameter was evaluated pre- and 6 months post-surgery. Continuous variables were presented as mean ± SD and compared with an unpaired *t*-test, while categorical data were expressed as percentages or numbers and compared with ʎ^2^ test.

A between-groups comparison examining the impact of pericardial approach and type of cardioplegia on right ventricular function over time was made with an analysis of variance and co-variance (ANOVA test) adjusted for patients’ age, sex, body surface area and right ventricular features basal values.

A *p* value < 0.05 was considered as statistically significant.

## Results

**Fig. 2:**
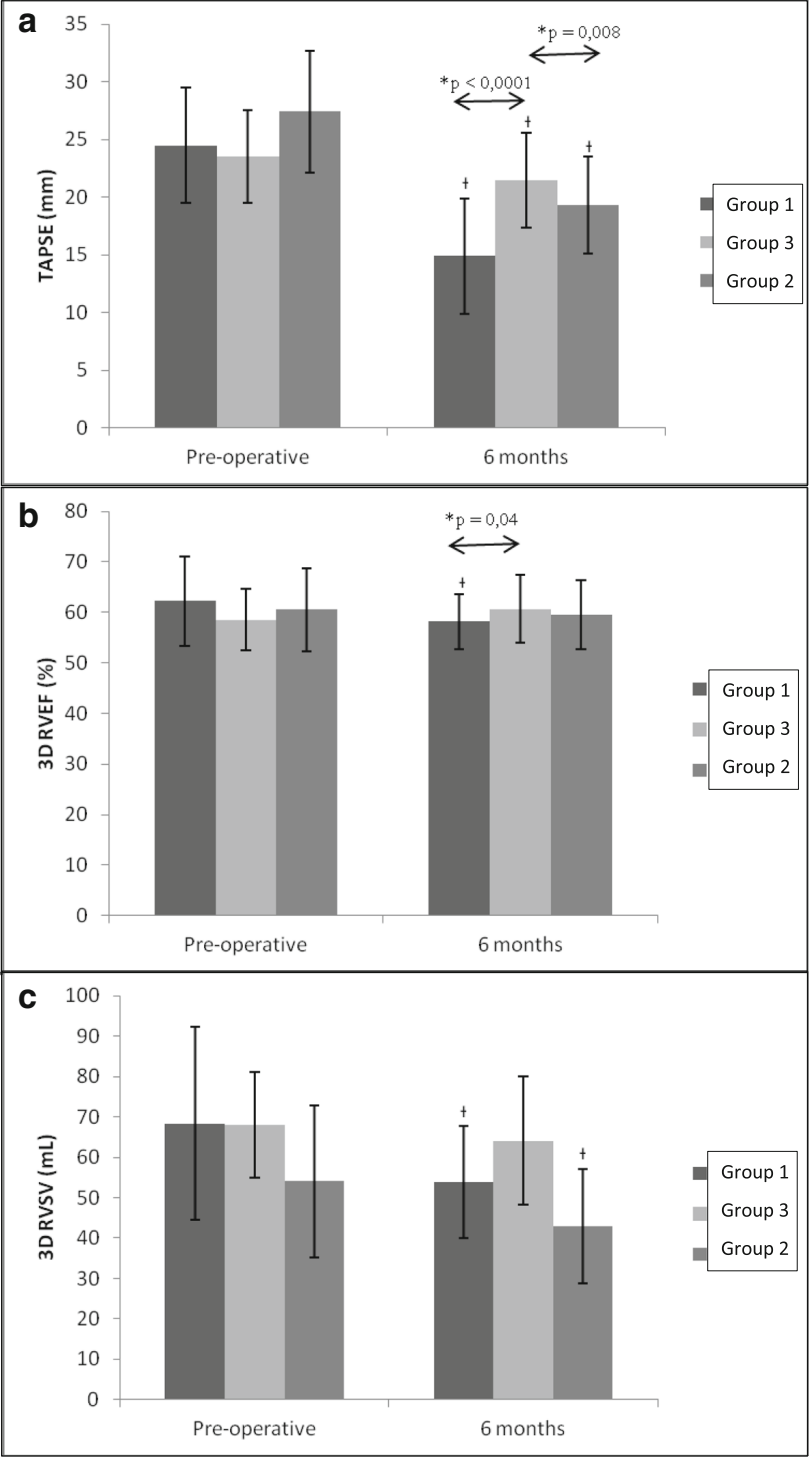
PANEL **a** - Mean tricuspid annular plane systolic excursion (TAPSE) and 95% confidence intervals (CIs) measured preoperatively and at 6 months postoperatively. * Between-groups comparison. ^Ɨ^ *p* < 0.05 vs preoperative; PANEL **b** - Mean three-dimensional right ventricular ejection fraction (3D RVEF) and 95% confidence intervals (CIs) measured preoperatively and at 6 months postoperatively. * Between-groups comparison ^Ɨ^ *p* < 0.05 vs preoperative; Panel **c** - Mean three-dimensional right ventricular stroke volume (3D RVSV) and 95% confidence intervals (CIs) measured preoperatively and at 6 months postoperatively. ^Ɨ^ *p* < 0.05 vs preoperative

All enrolled patients underwent mitral valve repair surgery without significant complications. After six month after surgery (185 ± 23 days), all cases had residual mitral regurgitation inferior to 1 degree (scale 1 to 4). At least one good quality, three-dimensional right ventricle dataset was acquired in all patients before surgery and six months after the surgery. Tables 4 and 5 and Panel A (Fig. 2) shows the mean values of the two-dimensional and three-dimensional parameters for each step of the study in both groups.

**Table 4:** Two-dimensional and three-dimensional echocardiographic parameters measured at pre- and 6 months post-surgery

Variable	Pre-surgery	Sixth month	P Within Group
TAPSE (mm)			
Group 1	24.5 ± 4.8	14.9 ± 3	< 0.0001
Group 2	27.4 ± 5.3	19.3 ± 4.2	0.0003
Group 3	23.5 ± 4	21.5 ± 4.1	0.013
SPAP (mmHg)			
Group 1	33.9 ± 4.8	25.7 ± 2.9	< 0.0001
Group 2	32.3 ± 5.1	28.3 ± 6.5	0.054
Group 3	34.9 ± 8.1	25.2 ± 3.1	0.049
RVEDV (ml)			
Group 1	111.7 ± 42.8	93.1 ± 26.2	0.016
Group 2	88.8 ± 26.1	72.2 ± 21.3	0.002
Group 3	116.5 ± 19.8	105.2 ± 20.6	0.039
RVESV (ml)			
Group 1	43.4 ± 22.5	39.2 ± 13.6	0.244
Group 2	34.7 ± 11.7	29.2 ± 9.2	0.048
Group 3	48.5 ± 11.6	41 ± 9	0.005
3D RVEF (%)			
Group 1	62.2 ± 8.9	58.2 ± 5.4	0.021
Group 2	60.5 ± 8.2	59.5 ± 6.9	0.579
Group 3	58.5 ± 6.1	60.7 ± 6.8	0.207
3D RVSV (ml)			
Group 1	68.3 ± 23.9	53.9 ± 13.9	0.004
Group 2	54.1 ± 18.8	43 ± 14.1	0.0039
Group 3	68 ± 13.1	64.1 ± 15.9	0.349

*TAPSE* Indicates tricuspid annular plane systolic excursion, *SPAP* Systolic pulmonary arterial pressure, *RVEDV* Right ventricular end-diastolic volume, *RVESV* Right ventricular end-systolic volume, *RVEF* Right ventricular ejection fraction, *RVSV* Right ventricular stroke volume

**Table 5:** Inter-group comparison of two-dimensional and three-dimensional echocardiographic parameters variations (6 months post-surgery versus pre-operative values)

Delta variable	Group 1	Group 2	Group 3	*P* Group 1 vs 3	*P* Group 1 vs 2	*P* Group 3 vs 2
TAPSE(mm)	−9.5	−8.1	−2	< 0.0001	0.12	0.008
SPAP(mmHg)	−8.1	−4	−9.7	0.66	0.86	0.65
RVEDV (ml)	−18.6	−16.5	−11.3	0.54	0.93	0.51
RVESV (ml)	−4.2	−5.5	−7.4	0.49	0.69	0.91
3D RVEF (%)	−3.9	−0.9	2.2	0.04	0.22	0.74
3D RVSV(ml)	−14.4	−11.1	−3.9	0.18	0.66	0.45
2D LVEF (%)	−7.6	−8.1	−3.8	0.08	0.84	0.19

TAPSE indicates tricuspid annular plane systolic excursion, SPAP systolic pulmonary arterial pressure, RVEDV right ventricular end-diastolic volume, RVESV right ventricular end-systolic volume, RVEF right ventricular ejection fraction, RVSV right ventricular stroke volume, LVEF left ventricular ejection fraction

Preoperative left and right ventricular function were in normal range for all patients. Basal TAPSE was slightly greater, but did not reach statistical significance, in traditional surgery (Group 1 and 2, respectively 24.5 and 27.4 versus 23.5 for mini-invasive patients; *p* = 0.17). All the three groups had similar preoperative 3D right ventricular function and cross-clamping/extracorporeal circulation time.

### Two-dimensional measurements

Postoperative TAPSE fall was found in each group (Table 4), but mini-invasive patients experienced a less marked variation (group 3: 21.5 ± 4.1 post- versus 23.5 ± 4 pre-; *p* = 0.01) compared to traditional surgery (Group 1 14.9 ± 3 post versus 24.5 ± 4.8 pre; *p* < 0.0001; Group 2 19.3 ± 4.2 post versus 27.4 ± 5.3 pre; *p* = 0.0003). The difference remained statistically significant after adjustment for patients’ age, sex, body surface area and basal TAPSE (Group 3 versus 1 *p* < 0.0001 and Group 3 versus 2 *p* = 0.008).

Systolic pulmonary arterial pressure showed a similar postoperative fall in each group.

Left ventricular volumes and ejection fraction decreased after surgery in a similar manner in all patients.

### Three-dimensional measurements

In Sterno-Buckberg patients (Group 1) end-systolic size-decreasing trend failed to reach statistical significance. However, in the other two groups (groups 2 and 3, Custodiol), both end-diastolic and end-systolic right ventricular volumes significantly diminished after surgery. Any inter-group size changes comparison showed a significant difference (Table 4).

In mini-invasive patients (group 3), right ventricular ejection fraction slightly augmented after surgery (60.7 ± 6.8 post versus 58.5 ± 6.1 pre; *p* = 0.2), while, in Group 1 decreased (58.2 ± 5.4 post versus 62.2 ± 8.9 pre; *p* = 0.02). In contrast, no significance difference was found in Group 2 (59.5 ± 6.9 post versus 60.5 ± 8.2 pre; *p* = 0.58). In addition, this variation was significantly different between mini-invasive versus sternotomy-Buckberg patients (*p* = 0.04). Similarly, right ventricular stroke volume diminished after surgery in all patients without significant inter-group differences.

## Discussion

The importance of RV function as an important physiopathology element in many different cardiovascular disease is a well-known phenomenon and it is an already validated prognostic index after cardiac surgery. In the past, obtaining an accurate postoperative evaluation of RV function has been made difficult by its complex anatomy and morphology. This problem was solved by the assessment of tricuspid annulus movement with 2D-Echocardiographic analysis that has been proved to be accurate, feasible, simple and reproducible in both normal and pathological patients [17]. The introduction of 3D-Echocardiography has permitted a big step forward in the evaluation of RV volume and function throughout the cardiac cycle [18, 19] permitting to calculate the right ventricular ejection fraction (RV-EF) that represents the global performance of the ventricle. The main limitation 3D-echocardiography is that is a technology usually available only in high-experienced center with skilled operators while 2D- TAPSE evaluation is an easy measurement for every echocardiographist.

Several studies report a reduction of TAPSE after cardiac surgery for congenital and acquired diseases [6, 20] without univocal explanation. Observing a post-operative TAPSE decrease, without variation in left ventricle function, has driven to interpret it as a simple modification with modest clinical impact. Many hypothesis have been presented in order to clarify this loss in RV performance measured at 2D-TTE, including cardiopulmonary bypass use [8, 21], geometrical changes of the right ventricular chamber (in association with interventricular septal paradoxical motion [22], intra-operative ischemia, right atrial injury due to cannulation procedure [23], poor myocardial protection [24], and extra myocardial causes (pericardial disruption, changes in Fossa Ovalis and post-operative adherence of the right ventricle to the thoracic wall, 9). The role of pericardial injury has also been highlighted in our precedent study [25]. One of the major confounding factors comparing traditional and mini-invasive surgery is represented by the use of single shot cardioplegia protocol in mini-MVR. To eliminate this bias and to evaluate myocardial protection impact on TAPSE we designed this three groups study.

To minimize all other possible confounding factors and to eliminate inter-operators difference, we acquired data only from patient underwent MVR by the same surgeon and who had pre- and postoperative TTE by one dedicated echocardiographist. Surgical valve repair technique doesn’t significantly differ within the 3 groups (Table 3) as well as the type of implanted prosthetic rings. Cross-clamping time and cardiopulmonary bypass time resulted comparable in all groups. The pericardial opening was entirely closed with a running suture in all patients.

TAPSE reduction was observed in all groups, but it has been significantly less marked in mini-invasive surgery group who underwent mini-anterolateral thoracotomy with supraphrenic lateral pericardial incision and Custodiol cardioplegia. Evaluating different cardioplegia protocol with same surgical approach (group 1 vs. 2) we did not find any significant differences. 3D-echocardiographic RV postoperative volume resulted comparable within the 3 groups. A slightly augmented RV ejection fraction was observed after mini-invasive surgery (group 3) while it decreased in patients underwent traditional median sternotomy despite the different cardioplegia protocol (group 1 Buckberg, group 2 Custodiol). According to these results, only different surgical approach impact RV function, in particular, lateral pericardial incision used in mini-invasive surgery is able to significantly limit right-ventricle longitudinal post-operative function decrease while the different type of cardioplegia protocol has no relevant effect on TAPSE.

To explain these findings more than a hypothesis can be provided. The first one could be that anterior pericardial incision modifies a portion of pericardium directly connected with the RV free wall while lateral opening, in the face of interatrial groove, does not interfere with RV motility. As we previously proposed [25], another explication could be linked to the shape itself of anterior pericardial incision. It consisted in a reversed T incision with a double opening line along the diaphragm that could modify the relationship between this muscle and the inferior RV wall leading to possible variations in longitudinal right ventricular contractile pattern and consequently to postoperative TAPSE fall. The different surgical repair techniques and prosthetic rings used to restore mitral valve competency and to assure long-term durability should not influence inter-group differences due to the homogenous distribution of them in the 3 groups. Different kind of resection or ring did not reach significant statistic difference within the study population (Table 3).

An additional important observation from this study is that, in parallel to the attended impairment of right ventricle long-axis function, also three-dimensional global systolic indexes showed a postoperative slight decreasing trend, which was less accentuated (or even inversed when talking about right ventricular ejection fraction) in mini-invasive group. In other words, it seems that, in our study, the right ventricle undergoes a kind of geometrical modifications too, which appear to be less affected by a lateral pericardial approach. Nevertheless, in our opinion, such tendency (which could be interpreted as clinically negligible since included in the normal range of measure variability) is partially different from what previously reported [3]. A prudent interpretation is justified by various considerations: 1) the increasing trend of right ventricular ejection fraction in mini-invasive group is not confirmed by a parallel stroke volume time-course; 2) the inter-group evaluation failed to show a statistically significant difference when comparing Group 3 vs Group 2 (thus perhaps suggesting a role played by cardioplegia too); 3) the intra-group three-dimensional variables variations were not significant in all groups.

Basing on these reasons, we believe that deeper investigations involving a greater patients’ population and a longer follow up time (which could elucidate that such trend was only a temporary phenomenon, as also pointed out by postoperative right ventricular ejection fraction fluctuations described by Tamborini et al.,3) are required before drawing definitive conclusions.

We studied a relative small number of cases. Despite this limitation and even though our data should be confirmed in a larger population, statistical analysis clearly defined significant changes and differences in the three groups, being able to highlight how different surgical protocols resulted in diverse right ventricular function postoperative trends.

### Study limitations

Main study limitation is represented by limited number of patients of the cohort analyzed and as well as the needed of longer echocardiographic follow-up. Larger population, different type of cardioplegia (Del Nido, St. Thomas, etc..) should be investigated.

## Conclusions

Minimally invasive mitral repair with lateral pericardial opening reduces postoperative TAPSE fall while cardioplegia protocol fails to have an impact onto longitudinal RV function. In our study, the right ventricle seems to show a clinically irrelevant geometrical modification too, whose entity appears to be less evident in case of lateral pericardial approach. These results could strengthen the use of minimally invasive approach also to preserve right ventricle function.

## Abbreviations

CBP: Cardiopulmonary bypass
IVC: Inferior vena cava
MVR: Mitral valve repair
TAPSE: Tricuspid annular plane systolic excursion
TTE: Transthoracic echocardiography
